# Simulated bacterial infection disrupts the circadian fluctuation of immune cells in wrinkle-lipped bats (*Chaerephon plicatus*)

**DOI:** 10.7717/peerj.3570

**Published:** 2017-08-03

**Authors:** Philipp Weise, Gábor A. Czirják, Oliver Lindecke, Sara Bumrungsri, Christian C. Voigt

**Affiliations:** 1 Department of Evolutionary Ecology, Leibniz Institute for Zoo and Wildlife Research, Berlin, Germany; 2 Department of Biology, Humboldt Universität, Berlin, Germany; 3 Department of Wildlife Diseases, Leibniz Institute for Zoo and Wildlife Research, Berlin, Germany; 4 Department of Animal Behaviour, Freie Universität Berlin, Berlin, Germany; 5 Department of Biology, Prince of Songkla University, Hat Yai, Thailand

**Keywords:** Acute phase response, Diurnal immune changes, Eosinophils, Neutrophils, Body mass loss, Leukocytosis

## Abstract

**Background:**

Leukocyte concentrations follow a circadian pattern in mammals, with elevated values at times of potential contact with pathogens and parasites. We hypothesized that this pattern is disturbed after an immune challenge.

**Methods:**

In Thailand, we captured wrinkle-lipped bats (*Chaerephon plicatus*), when they returned to their colony at dawn. We challenged half of the animals (experimental group) with bacterial lipopolysaccharides and treated the others only with the carrier liquid (control group). We then compared body mass changes and differences in circulating immune cell counts at 8 h post-treatment.

**Results:**

In experimental animals, we observed an increase in total leukocyte and neutrophil numbers of 17% and 95%, respectively. In control animals, concentrations of leukocytes decreased by 44% and those of neutrophils remained constant. Experimental treatment had no effect on lymphocytes, yet changes in eosinophil numbers were explained by sex. Eosinophils decreased by 66% in females and by 62% in males. Basophils and monocytes were rarest among all observed cell types and analysis was either impossible because of low numbers or yielded no significant effects, respectively.

**Discussion:**

Our findings show that a simulated bacterial infection triggered a neutrophil-associated immune response in wrinkle-lipped bats, indicating a disruption of the diurnal fluctuation of immune cells. Our study suggests that bats exhibit circadian rhythms in immune cell counts. The magnitude of these fluctuations may vary across species according to specific-specific infection risks associated with colony sizes or specific roosting habits.

## Introduction

Considering the direct involvement immunity has on survival and fitness, evolution should favor a highly developed immunocompetence. However, mounting and maintaining an immune response is considered to be costly both in terms of energy and pathology, thus being traded against other relevant life-history traits such as reproduction and growth (Lochmiller & Deerenberg, 2000; Sheldon & Verhulst, 1996). Accordingly, organisms should optimize their immunity not only in relation to their life-history traits, but also in response to changing environments. As a result, immunity is known to vary on a seasonal and daily scale to respond efficiently to the variation in climatic conditions, food availability and risk of infection (Pap et al., 2010; Scheiermann, Kunisaki & Frenette, 2013; Labrecque & Cermakian, 2015). Immunocompetence should be most pronounced when animals face the highest risk of getting exposed to novel pathogens or parasites, i.e., immunity should be best prepared for daytime activity of diurnal animals and for nighttime activity of nocturnal animals. Previous work in humans and rodent models showed that indeed both humoral and cellular immune components display circadian dynamics (Scheiermann, Kunisaki & Frenette, 2013) which have been linked with circadian response to acute inflammatory challenges (Halberg et al., 1960) and differences in disease manifestations (Labrecque & Cermakian, 2015).

In humans, leukocyte counts exhibit a daily maximum and a nightly minimum (Málek et al., 1962), however the peak of each subtype of immune cell varies in time. While the numbers of agranulocytes, such as monocytes, T and B-cells reach a maximum during sleep (night), neutrophils, natural killer cells and activated T-cells peak during the active phase (daytime) (Born et al., 1997). It has been shown that the functions of some of these cells also follows a circadian rhythm, for example reactive oxygen species-producing capacity of neutrophils is activated during daytime (Shiraishi et al., 1996), but IL-2 secretion peaks during night (Born et al., 1997). Despite their nocturnal activity, laboratory rodents show similar circadian rhythms in immune cell counts as humans (Dhabhar et al., 1994; Scheiermann, Kunisaki & Frenette, 2013), indicating well conserved molecular and biochemical control of these patterns. Diurnal changes in leukocyte counts are mediated by neural (central and peripheral clock) and endocrine (e.g., corticosterone and adrenal catecholamines) factors, which play a crucial role in immune modulation (Dhabhar et al., 1994; Widmaier & Kunz, 1993). Further, the extent of circadian rhythm of immune functions may vary between strains and sex in laboratory rats (Griffin & Whitacre, 1991). However there is limited information on the circadian rhythms of the immune system in wildlife, information which is crucial taking into account the immune differences between captive and free-living animals (Abolins et al., 2011; Boysen, Eide & Storset, 2011; Tian et al., 2015a).

In free-living birds, immunological parameters also vary with daytime (Ots, Murumagi & Horak, 1998; Navarro et al., 2003). However, the patterns should be specific for species and dependent on the immune effectors assessed (Zylberberg, 2015). Similar to humans and lab rodents, breeding female great tits (*Parus major*) show higher total leukocyte levels at the end of the active period, yet this is due to both elevated numbers of lymphocytes and neutrophils (Ots, Murumagi & Horak, 1998). In house sparrows (*Passer domesticus*), T-cell mediated response to a challenge showed maximum response 6 h post-treatment, yet this varied between day and night time (Navarro et al., 2003).

In this study, we were interested in how the cellular immunity of a wild mammal varies with daytime and if diurnal fluctuations are disrupted when animals are challenged. We used wild bats as our model organism, since bat immunology is particularly interesting owing to the potential of high pathogen load in bats (Luis et al., 2013) and because of the seemingly asymptomatic disease dynamics when bats contact a pathogen (Kuzmin et al., 2011). Bats form the second largest mammalian clade, exceeded in species richness only by Rodentia (Tsang et al., 2016). They are ecologically diverse, inhabit a multitude of ecological niches worldwide and therefore display a great number of different life-history traits. Bats are known or suspected reservoirs for many emerging pathogens like Nipah (Yob et al., 2001), Hendra (Halpin et al., 2000), Ebola (Leroy et al., 2005) and other viruses (reviewed in Wang & Cowled, 2015). Although the study of bat immunity has gained momentum over the past years (especially immunogenetics and molecular immunology) (Baker & Zhou, 2015), we still miss a detailed knowledge of the intrinsic and extrinsic factors that influence the immunocompetence of bats. Allen et al. (2009) demonstrated an association of roost environment and colony size with immune parameters in Brazilian free-tailed bats (*Tadarida brasiliensis*). Schneeberger, Czirják & Voigt (2013a) showed that species-specific immune phenotype is linked to roosting type and diet in Neotropical bats. Further, a study by Christe, Arlettaz & Vogel (2000) in greater mouse-eared bats (*Myotis myotis*) suggested that the reproductive stage of female bats, particularly pregnancy, influences the cellular immunocompetence. Greiner et al. (2010) investigated how stress and sexual hormones interplay with the cellular immune response. Most of these past field studies focused on cell-mediated immunocompetence (Christe, Arlettaz & Vogel, 2000; Allen et al., 2009; Greiner et al., 2010), while recently bat acute phase response received attention (Schneeberger, Czirják & Voigt, 2013b; Stockmaier et al., 2015; Otálora-Ardila et al., 2016). Latter studies administrated lipopolysaccharides (LPS) and characterized the acute phase response via leukocyte counts in 24 h post-injection (Schneeberger, Czirják & Voigt, 2013b; Stockmaier et al., 2015), ignoring the possibility that the immune challenge may also disrupt the diurnal fluctuation in cellular immunity. This is a critical point and partly might explain the observed discrepancies between the studies (Schneeberger, Czirják & Voigt, 2013b; Stockmaier et al., 2015; Otálora-Ardila et al., 2016), as the strength of the response to acute inflammatory challenges depends on the time of immune challenge (Halberg et al., 1960) and the LPS-associated leukocytosis (e.g., increase in leukocyte numbers) disappears 24 h post-injection in humans (Richardson et al., 1989).

In our study, we used wrinkle-lipped bats (*Chaerephon plicatus*) (Buchannan 1800; Molossidae) as our model species. *Chaerephon plicatus* is a cave dwelling bat species that is relatively abundant in Southeast Asia, including Thailand (Hillman, 2006). The species is of economic relevance to local communities and even national economy owing to the production of guano as organic fertilizer and the consumption of insects that are a pest to rice plants (Leelapaibul, Bumrungrsi & Pattanawiboon, 2005; Wanger et al., 2014). Caves may count several million individuals, yet colonies have experienced significant declines over the past decade (Utthammachai et al., 2008; Hillman, 2006). The aim of our study was to provide first insights into the eco-immunology of *C. plicatus*, by investigating whether the cellular immune phenotype and acute phase response is influenced by sex, body condition and the time of day. In order to achieve this, we collected baseline values of total and differential white blood cell counts and then quantified the cellular immune response of bats at 8 h after injecting LPS or PBS (see below). We were aiming to conduct these experiments in non-reproducing females and males, yet at our study site female *C. plicatus* were at the stage of late lactation. Since sex and reproductive state are known to influence the immune response (Sheldon & Verhulst, 1996; Lochmiller & Deerenberg, 2000; Pap et al., 2010), we could not separate between these two effects. However, previous research in bats confirmed that lactating bats are not necessarily less immunocompetent than non-reproductive females (Christe, Arlettaz & Vogel, 2000). Thus, we assume in our study that the immunity of lactating *C. plicatus* is similar to that of non-reproducing females. Accordingly, we predicted that the response in cellular immunity differs between female and male *C. plicatus*. Further, we expected that bats from the control group (see below) would show an increased cellular immunity in morning hours when fed bats return to their daytime roost and low levels after having rested for several hours if potential pathogen exposure during foraging is the driving force for diurnal fluctuations of immune parameters and the opposite pattern if potential pathogen exposure in the roost is the driving force. Further, we predicted that a poor body condition would lower the immune response when bats are challenged. Finally, we expected that diurnal fluctuations is disturbed in LPS challenged bats, especially in case of neutrophils, since they constitute the first line of defense against bacterial infections.

## Material and Methods

### Field sampling and experimental procedure

The wrinkle-lipped free-tailed bat (*Chaerephon plicatus*) occurs in 18 major colonies in Thailand, all located in caves (Wanger et al., 2014). *Chaerephon plicatus* has two breeding seasons per year, with most pregnancies occurring in February–March and August–September (Utthammachai et al., 2008). Our study was conducted in early November 2015 at the Wat Khao Wongkot bat-cave, located approximately 160 km north of Bangkok (province Lop Buri; 15.01814459°; 100.54520403°). The experiments complied with the current laws of Germany and Thailand, and were performed as part of the permit #0002/4508 granted by the National Research Council of Thailand (NRCT) and as part of the permit #108/59 granted by the Department of National Park, Wildlife and Plant Conservation (DNP) and were approved by the animal welfare and ethics committee of the Leibniz Institute for Zoo and Wildlife Research.

The local *C. plicatus* colony is estimated to include more than one million individuals (S. Bumrungsri, pers. comm., 2016). Study animals (*n* = 44, 20 adult males and 24 adult females) were captured between 8:00 a.m. and 11:00 a.m inside the cave by picking them from the walls of the cave. Out of the 24 females, we categorized 12 as lactating at a late stage and 12 as early post-lactating adults by examining the appearance of nipples and the surrounding pelage. For further analysis, we lumped data of the two female groups since we assumed that the transition from lactation to post-lactation is gradual and that the immunology of females lactating at a late stage was similar to those at an early post-lactating stage.

The animals were randomly divided in two groups: bats of the experimental group were injected intraperitoneally with 34 µl of 1 mg ml^−1^ LPS from *E.coli* O111:B4 (# L2630; Sigma-Aldrich, Munich, Germany) diluted in phosphate buffered saline (PBS; pH = 7.3, Carl Roth, Karlsruhe, Germany) using a sterile disposable syringe, while each animal of the control group received 34 µl of PBS. Used antigen concentrations were similar to those reported in previous experiments in bats (Schneeberger, Czirják & Voigt, 2013b; Stockmaier et al., 2015). After injection, all animals were transported to a maintenance facility, where they were kept undisturbed in individual cotton bags until the evening. From each bat, we collected a blood sample before injection and 8 h post-injection (see below). We applied the same treatment duration to all animals (±20 min) to warrant comparability. We chose a time of 8 h post-injection because of several reasons. First, we were interested in diurnal fluctuations and thus we expected the largest effect in samples that were collected between 6 and 12 h after the emergence of bats. Second, previous studies used an antigen exposure time of about 12 h in a related species (Allen et al., 2009) and a study on the temporal dynamics of leukocyte mobilization and infiltration documented a strong response after about 6–12 h (Turmelle et al., 2010). Third, we refrained from keeping bats longer than the selected 8 h post-injection time, because the corresponding time of day coincided with the emergence of bats. We assumed that preventing bats from emerging to their foraging areas would cause acute stress in animals, which could have biased the outcome of our experiment.

During both time points, we measured the body mass with a spring balance (Pesola; Schindellegi, Switzerland; accuracy 0.1 g) and forearm length with a caliper (accuracy 0.1 mm). We calculated body condition by dividing body mass by forearm length (Schneeberger et al., 2014). For blood collection, we wrapped gently a cotton bag around the body of a bat and thus mechanically restrained the animal so that blood could be drawn from the antebrachial vein of the right wing (see below). All animals were released at the site of capture after the second blood sample was taken.

### Hematological analysis

From each individual, we obtained a small blood sample (approximately 125 µl) by puncturing the antebrachial vein with a sterile needle and then transferring the blood to three heparinized micro hematocrit capillaries (Carl Roth, Karlsruhe, Germany). One drop of blood was used to prepare a blood smear, the remaining sample was transferred to a 1 ml Eppendorf tube (Eppendorf, Hamburg, Germany) and stored on ice until later analysis. Total leukocyte numbers were measured for all samples using the classical haematological method (Tian et al., 2015b). Ten µl of blood sample was diluted 1:20 in Turk’s solution (1% acetic acid: Carl Roth, Karlsruhe, Germany; crystal violet: Merck, Darmstadt, Germany) and after the acid lysed the red blood cells, an aliquot of 20 µl was transferred into the Neubauer chamber (Carl Roth, Karlsruhe, Germany) and examined under 200× magnification using an optical microscope (Carl Zeiss, Jena, Germany). The cell numbers were counted in a total area of 4 mm^2^ and the total leukocyte number was calculated taking into account the dilution factor and the depth of the chamber. To obtain the number of each leukocyte type, blood smears were fixed in methanol and stained according to Leishman’s staining protocol. Hemograms were performed counting 100 leukocytes under a 1,000× magnification under a microscope (Olympus, Tokyo, Japan). The proportions of the different leukocyte cell types (lymphocytes, neutrophils, eosinophils, basophils and monocytes) were calculated. All immune cell counts are reported as cells per µl blood (µl^−1^).

### Statistical analysis

Statistical analyses were done either using SYSTAT (vs 11, SYSTAT Software GmbH; Erkrath, Germany) or the open source software R (R Development Core team, 2013). Specifically, we used packages “mass” and “car” (Venables & Ripley, 2002; Fox & Weisberg, 2011). We calculated the differences in total and differential leukocyte counts between first and second sampling event. Then we compared these differences between experimental and control group. We chose one-way ANOVA with delta values as the response variable and treatment, sex and interaction between treatment and sex as factors. One female individual of the control group had a highly elevated total leukocyte count of 24,500 cells µl^−1^ and its eosinophil granulocyte count was more than threefold higher compared to those of conspecifics. We assumed that this individual acquired recently a parasite infection and, thus, we excluded it from further statistical analysis. Basophils were too rare to be considered in this analysis.

The statistical model requirements were fulfilled for leukocytes, lymphocytes, neutrophils and eosinophils. To achieve normal distribution of the delta monocyte values, the data was transformed using the quantile function of the standard normal distribution, i.e., the probit function. Negative values were first ranked and subsequently divided by twice the maximum rank plus one, to achieve a distribution within the boundaries 0 and 0.5. Zeros were set to 0.5. Ranked positive values plus one were divided by the maximum rank plus one and subsequently divided by two, to achieve a distribution within the boundaries of 0.5 and 1. Finally, the values were transformed using the probit function and normal distribution was achieved. The significance level was set to α = 0.05 for all analysis. Parameters are presented as means ± one standard deviation if not stated otherwise.

## Results

Before the experimental treatment, males weighed on average 17.8 ± 1.4 g and females 17.2 ± 1.1 g, which was not significantly different (*t*_42_ = 1.48, *P* = 0.148). Baseline WBC levels are reported in Tables 1 and 2. Baseline parameters did not differ between sexes (Mann–Whitney *U*-Test, *P* > 0.05), except for eosinophils (*P* = 0.009). This pattern was also true for relative proportions of leukocyte subsets, where only percentage contributions of eosinophils to total leukocytes proved different between sexes (Mann–Whitney *U*-test, *P* = 0.002).

**Table 1:** Total- and sex-specific baseline values (Mean ± SD, range) of different leukocytes for wrinkle-lipped bats (*Chaerephon plicatus*).

	Leukocyte/µl blood	Neutrophil/µl blood	Basophil/µl blood	Eosinophil/µl blood	Lymphocyte/µl blood	Monocyte/µl blood
**Total**	7,626 ± 2,540	3,494 ± 1,613	13 ± 44	840 ± 611	3,149 ± 1,667	130 ± 155
(*n* = 43)	2,450–14,600	8,591–1,173	0–249	0–2,576	760–7,763	0–774
**Males**	8,058 ± 3,007	3,717 ± 1,833	9 ± 29	567 ± 356	3,670 ± 1947	94 ± 104
(*n* = 20)	2,450–14,600	1,397–8,591	0–133	0–1,515	760–7,763	0–350
**Females**	7,241 ± 2,047	3,301 ± 1,406	16 ± 54	1,077 ± 691	2,696 ± 1,252	161 ± 186
(*n* = 23)	4,150–12,150	1,173–7,452	0–249	146–2,576	1,196–6,197	0–774

**Table 2:** Total- and sex-specific baseline values (Mean ± SD, range) of the relative numbers of different leukocyte subsets in wrinkle-lipped bats (*Chaerephon plicatus*).

	Neutrophil (%)	Basophil (%)	Eosinophil (%)	Lymphocyte (%)	Monocyte (%)
**Total**	46 ± 14	0 ± 1	7 ± 11	41 ± 13	2 ± 2
(*n* = 43)	20–81	0–3	0–32	13–69	0–12
**Males**	47 ± 13	0 ± 1	4 ± 7	44 ± 14	1 ± 2
(*n* = 20)	26–71	0–3	0–13	25–69	0–5
**Females**	46 ± 15	0 ± 1	8 ± 15	37 ± 12	2 ± 3
(*n* = 23)	20–81	0–3	2–32	13–67	0–12

**Figure 1:**
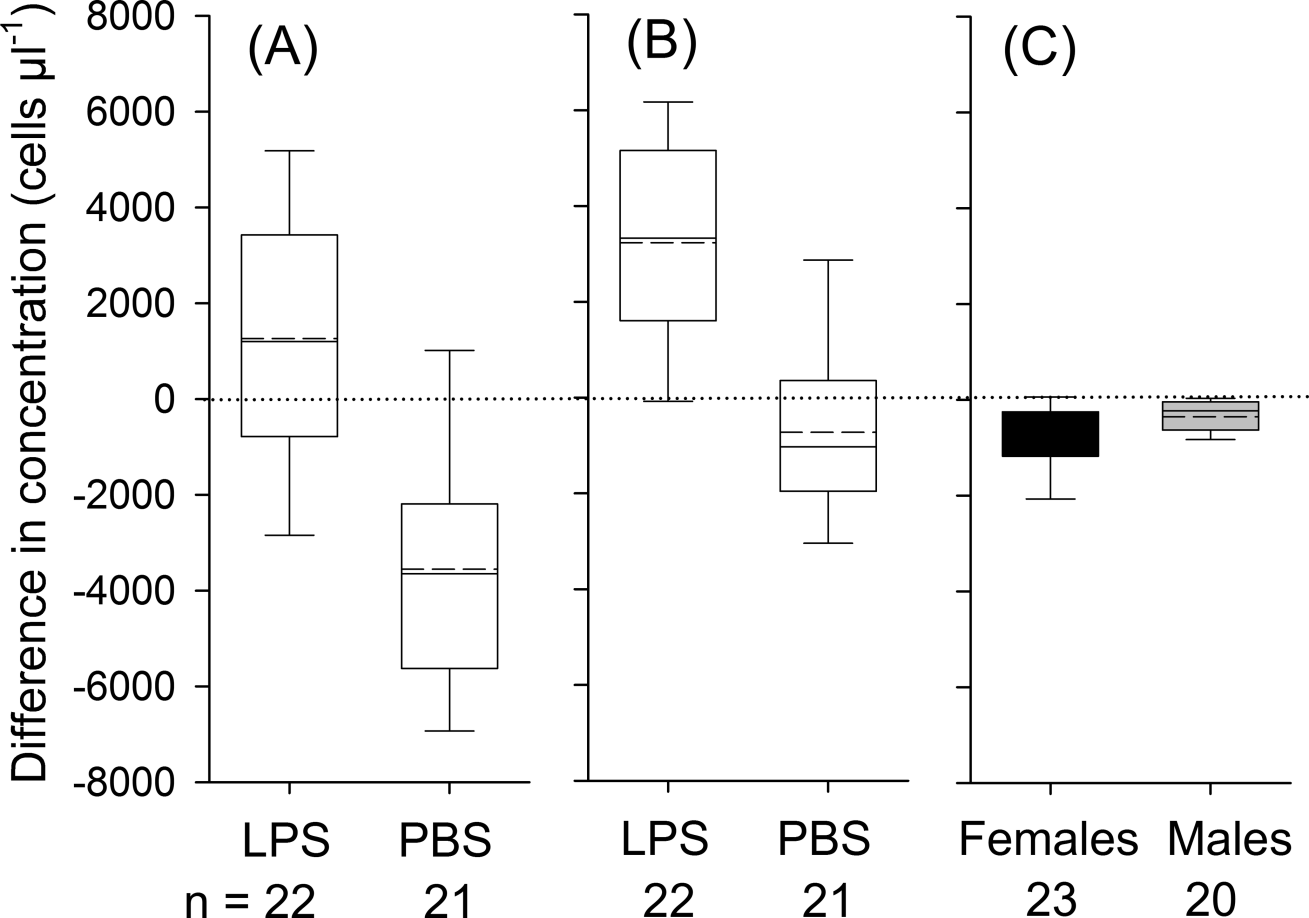
Treatment effects for leukocytes (A; treatment: LPS vs. PBS), for neutrophils (B; LPS vs. PBS) and eosinophils (C; males vs. females; black boxes indicate data from female and gray boxes data from male bats). Whiskers range until the last value that is within the 1.5 × interquartile range. The solid black line indicates the median and the dashed line mean values. The effect of treatment (LPS vs. PBS) or sex on variation in differences in concentrations was calculated based on one-way ANOVAs (two-tailed). Only significant effects at *P* <0.05 are depicted in the graph. Data refers to individual data points, without any replicates per individual.

Bats decreased in body mass by on average 1.1 ± 0.8 g during the experimental period, yet body mass loss was independent of treatment (*t*_41_ = 0.85; *P* = 0.40). We detected a significant influence of treatment, but not of body condition, sex or the interaction between treatment and sex on changes in leukocyte concentrations (Table 3). In LPS treated animals, leukocytes increased 8 h post-injection on average by 1,263 ± 2,842 cells µl^−1^(17% increase in relation to baseline values; *t*_21_ = 2.1, *P* = 0.049), whereas in the control group, the number of leukocytes decreased by 3,750 ± 2,677 cells µl^−1^ (44%; Fig. 1A; *t*_21_ = 6.1, *P* < 0.001).

**Table 3:** Results of analysis of variance in immunological parameters based on sex, treatment as categorical variables (including their interaction) and body condition as a co-variable (degrees of freedom were 1,38 for all tests; significant effects are highlighted in bold and trends (0.1 > *p* > 0.05) are highlighted in italic and bold).

Blood parameter	Sex	Treatment	Sex × Treatment	Body condition
Leukocytes	*F* = 1.1, *P* = 0.31	*F* = 29.5, ***P*** < **0.001**	*F* = 1.5, *P* = 0.23	*F* = 0.6, *P* = 0.46
Lymphocytes	*F* = 2.5, *P* = 0.12	*F* = 2.3, *P* = 0.14	*F* = 0.2, *P* = 0.66	*F* = 0.022, *P* = 0.88
Neutrophils	*F* = 2.7, *P* = 0.11	*F* = 30.8, ***P*** < **0.001**	*F* = 0.70, *P* = 0.41	*F* = 0.513, *P* = 0.48
Basophils	na	na	na	
Eosinophils	*F* = 4.6, ***P*** = **0.04**	*F* = 1.0, *P* = 0.31	*F* = 3.2, ***P*** = **0.08**	*F* = 0.267, *P* = 0.61
Monocytes	*F* = 1.1, *P* = 0.310	*F* = 0.01, *P* = 0.92	*F* = 0.20, *P* = 0.65	*F* = 0.45, *P* = 0.51

For neutrophils, the analysis showed a significant effect of treatment, but not of body condition, sex or the treatment × sex interaction on the relative change in corresponding cell numbers (Table 3). In LPS treated animals, concentrations of neutrophils increased by 3,234 ± 2,489 cells µl^−1^ (95%; *t*_21_ = 6.1, *P* < 0.001) and in PBS treated animals neutrophils remained constant (*t*_21_ = 1.71, *P* = 0.103, Fig. 1B). For lymphocytes, we found no significant effect of sex, body condition, treatment or interaction between sex and treatment on variation in lymphocyte numbers (Table 3). On average, lymphocyte counts dropped by 1,751 ± 1,445 cells during the experimental period (*t*_42_ = 7.9, *P* < 0.001). In eosinophils, we found an effect of sex and a trend for an effect for the interaction between treatment and sex on changes in concentration (Table 3). In females, the total number of eosinophils decreased by 706 ± 625 cells µl^−1^ (66%; *t*_22_ = 5.4, *P* < 0.001), whereas in males the number of eosinophils decreased by 352 ± 400 cells µl^−1^ (62%; *t*_19_ = 3.93, *P* = 0.001; Fig. 1C). Monocytes were found in about 90% of all samples. After data transformation, the ANOVA revealed no significant effect for any of the factors on monocytes (Table 3). Across all animals, monocytes decreased during the experimental period (*t*_42_ = 3.1, *P* = 0.004).

## Discussion

To our knowledge this is the first study investigating the diurnal fluctuations of immunity in a wild mammal. Further, it is the first study in a wild mammal that addresses the question if diurnal fluctuations are disrupted after a simulated bacterial infection. Specifically, we asked if immune challenged individual bats maintain a high number of circulating immune cells during daytime. Bats from the experimental group responded with neutrophil-dominated leukocytosis 8 h post-injection, whereas the overall leukocyte numbers decreased in the control group during the same period, indicating that LPS induces an innate cellular response in this bat species. As expected for animals of the control group, almost all immune cell counts decreased over the daytime, except for neutrophil granulocytes. Moreover, contradictory to the study by Christe, Arlettaz & Vogel (2000), the measured immune response did not differ between the sexes, except for eosinophil counts which differed between males and females, yet irrespective of treatment.

The mean absolute leukocyte count (7,626 ± 2,540 cell µl^−1^) in *C. plicatus* is among the highest values reported in Yangochiropteran species (see Valdivieso & Tamsitt, 1971; Olsson, 2009; Schinnerl et al., 2011). The values are similar to those documented for the Neotropical bat *Trachops cirrhosus* (Schinnerl et al., 2011), yet the two species differ in their immune cell subtype compositions. The exceptionally high leukocyte counts in *C. plicatus* contradicts at least partly the study by Schneeberger, Czirják & Voigt (2013a), who found a correlation between WBC counts and feeding habits in Neotropical bats. Specifically, Schneeberger and colleagues showed that insectivorous species had the lowest WBC counts compared to phytophagous and carnivorous species. However, Schneeberger, Czirják & Voigt (2013a) did not include colony size or sociality of bat species in their model. Epidemiological theory predicts that disease risk increases with group size as a result of high contact and increased transmission rates (Nunn, Gittleman & Antonovics, 2003). Accordingly, highly social species should invest more in their immunity as it has been shown in carnivore, but not in primate species (Nunn, 2002; Nunn, Gittleman & Antonovics, 2003). Taking into account the extremely highly gregarious behavior of this bat species, we argue that the high immune cell numbers in *C. plicatus* are driven by the species’ social behavior and roosting ecology. Further intra-specific and comparative studies are required to test this hypothesis. The main circulating immune cell type in bats is lymphocytes (Schinnerl et al., 2011; Valdivieso & Tamsitt, 1971; Olsson, 2009; Schneeberger, Czirják & Voigt, 2013a). However, differential blood cell counts of free-tailed bats (Molossidae), including *C. plicatus,* are dominated by neutrophils. Additionally, we reported particularly high eosinophil counts in this species, which might be due to the high number of ectoparasites observed in the specific colony (own obs.).

We assumed that bats would show a reversed diurnal fluctuation in cellular immunity compared to humans, if diurnal fluctuations are caused by potential pathogen exposure during foraging. Accordingly, we predicted high levels in morning hours when bats return fed to their daytime roost and low levels after having rested and fasted for several hours. Indeed, all immune cell counts, except for neutrophils, decreased over the daytime in control bats. This contradicts results in humans where the peak of each leukocyte subtype varies in time (Born et al., 1997). Further studies on the circadian dynamics of bats’ cellular and humoral immunity with a better temporal resolution are warranted, including challenges at different periods of the day to understand whether disease outcome presents a similar fluctuation as it was described in mice (Halberg et al., 1960) or if the time of the challenge might explain the species-specific acute phase differences between bat species (Schneeberger, Czirják & Voigt, 2013b; Stockmaier et al., 2015; Otálora-Ardila et al., 2016). Moreover, time of blood sampling needs to be considered when studying the immune system of free-living mammals in order to make data comparable across studies.

An alternative explanation for the observed decrease of cellular immune parameters in the control group would be that the observed patterns are associated with acute stress. In mammals, stress imposed on animals by, e.g., handling and captivity, may affect the abundance and composition of leukocytes (Dhabhar et al., 1994; Widmaier & Kunz, 1993; Davis, Maney & Maerz, 2008). A study by Widmaier & Kunz (1993) has shown for captive bats that handling and bleeding times beyond 3 min causes significant increases in plasma cortisol and glucose levels compared to resting individuals. These stress-induced endocrine changes are known to alter the leukocyte number as well as the distribution of leukocytes between numerous immune compartments and the blood (Dhabhar et al., 1994; Widmaier & Kunz, 1993; Davis, Maney & Maerz, 2008). However the stress-induced endocrine changes affect the leukocyte subtypes in different ways, e.g., cause decrease in lymphocytes and increase in neutrophils (Davis, Maney & Maerz, 2008). Since almost all cell types showed a decrease, we think that this is an unlikely explanation for the observed pattern in *C. plicatus*. Besides, all animals were treated in the same way and thus they were facing similar levels of stress, yet decreases of cellular immune parameters were mostly observed in control animals. A second alternative hypothesis is that the observed changes in the control group are associated with daily torpor. Many bats lower their body temperature and metabolic rate on a daily base by entering a torpid state. Although we have no information if *C. plicatus* uses torpor on a daily scale, or how torpor (daily or hibernation) influences the circulating immune cell counts in bats, the most prominent change in the torpid immune system is leuocopenia (e.g., decrease in leukocyte numbers) with both granulocytes (neutrophils, eosinophils and basophils) and agranulocytes (monocytes and lymphocytes) being affected (Bouma, Carey & Kroese, 2010). Torpid animals also show reduced inflammatory and acute phase response: hypothermic animals respond to LPS challenges only when they returned to normothermic temperatures during the post-challenge arousals (Prendergast et al., 2002). As our experimental animals clearly showed an acute phase response (see above), we exclude the possibility of a torpor-associated decrease in circulating leukocytes. A not mutually exclusive explanation for the observed leukocyte patterns in the control group is based on the assumption that the initial WBC count was already elevated compared to baseline levels and caused by previous food intake. The animals were bled immediately after they returned to the colony at the end of the night. Therefore, consumed prey may not have been fully digested. Food intake is accompanied by a high load of antigens that require host defense, which might explain the observed pattern. For example, humans show an increase in WBC about 1 h–2.5 h after the ingestion of food (Hansen et al., 1997; Lippi et al., 2010).

Interestingly, the immune-challenged bats did not loose significantly more body mass than animals of the control group. This contradicts the results of previous LPS challenge experiments in Neotropical bat species (Schneeberger, Czirják & Voigt, 2013b; Stockmaier et al., 2015) where body mass of experimental animals decreased, most likely as a result of reduced food intake after LPS induced sickness behavior, a phenomenon previously described in other mammals (Kozak, Conn & Kluger, 1994). The contrasting findings in these studies are probably the result of different experimental designs. While Schneeberger, Czirják & Voigt (2013b) and Stockmaier et al. (2015) kept the animals for 24 h, a recent study by Otálora-Ardila et al. (2016) kept the animals only for 11 h. However all previous studies provided food and water *ad libitum* or force fed the animals*,* while in our study animals were kept during daytime for 8 h without food and water, because *C. plicatus* does not feed on insects or drink when roosting in caves. The prolonged experimentation time and the possibility of food intake very likely increased the mass differences between the treatment groups in previous studies. Additionally, our animals were kept in captivity during their natural resting phase, a period which naturally would be associated with a decrease in body mass (as seen in the control group). Moreover, the energetic trade-off between different activities (e.g., hunting, parental care, avoiding predation) and immune response of the active period might decrease or disappear during the resting phase, thus the energy saved in this period could be allocated towards immunity without using resources and causing decrease in body mass.

Our findings suggest that the immune challenged bats disrupted, at least partially, the circadian fluctuation of immune cell counts observed in control group, i.e., LPS treated animals showed an increase in total leukocytes (leukocytosis) and also in neutrophils (neutrophilia) relative to the morning baseline values. The leukocytosis described in the experimental group is in line with results of LPS challenge experiments conducted in other mammals, such as Seba’s short-tailed bats, *Carollia perspicillata* (Schneeberger, Czirják & Voigt, 2013b) and mice (Copeland et al., 2005), but contrasts the findings in a closely related species, Pallas’s mastiff bats (*Molossus molossus*) (Stockmaier et al., 2015). Similarly to the differences observed in the case of body mass changes, this discrepancy in leukocytosis between the three bat studies might be caused by differences in the experimental design (e.g., route of administration, experimentation time). Depending on the pharmacokinetics of the injected substances, the administration route can lead to differences in the timing and the quality of reaction (Turner et al., 2011). The above mentioned bat studies used different routes of administration for the LPS: it was injected intraperitoneally in *C. plicatus* and subcutaneously in *Carollia perspicillata* and in *M. molossus* (this study; Schneeberger, Czirják & Voigt, 2013b; Stockmaier et al., 2015). Additionally, the LPS-associated leukocytosis disappears 24 h post-injection in humans (Richardson et al., 1989), thus it might not be surprising that there are differences between our and the study in Pallas’s mastiff bats (Stockmaier et al., 2015). However, Seba’s short-tailed bats subcutaneously challenged with LPS show leukocytosis even 24 h after the challenge (Schneeberger, Czirják & Voigt, 2013b), suggesting that a prolonged response may have evolved in some other bat species to deal with infections.

Neutrophils are the main circulating immune cells in *C. plicatus*, thus it is not surprising that the LPS-associated leukocytosis is characterized by neutrophilia. Neutrophils constitute the first line of defense against bacterial infections and are the primary phagocytic cells that rapidly proliferate in response to infections and inflammation (Davis, Maney & Maerz, 2008; Murphy & Weaver, 2016). In early stages of infection, neutrophils migrate to affected regions where they are essential in the fight against infections (Shiraishi et al., 1996; Turmelle et al., 2010). The increase of neutrophils in the peripheral blood is usually caused by demargination from the endothelium and reduced apoptosis in reaction to an immune response (Zahorec, 2001). LPS administration in humans is characterized by marked changes in leukocyte subsets consisting of monocytopenia (1–3 h post injection), lymphocytopenia (1–6 h post injection) and a nearly granulocytopenia (neutropenia, 1 h post injection) that promptly reverses to a granulocytosis (neutrophilia, 2–6 h) (Richardson et al., 1989). All these changes are driven by the dynamics of plasma epinephrine and cortisol levels and all immune cell counts are restored 24 h after the injection (Richardson et al., 1989). In contrast to humans, our LPS treatment had no effect on other immune cell subsets (lymphocytes, monocytes or eosinophils), which followed the same diurnal patterns as control bats. These results indicate (1) that in bats the leukocyte subset changes associated with LPS challenges are not mediated by an acute adrenocortical response, and (2) the physiology and function of neutrophils might be different in bats compared to other mammals.

Except for eosinophils, we did not observe any sex differences in baseline immune cell parameters, neither on the absolute nor on the relative scale. Also, we did not see any sex-specific differences in diurnal changes of leukocyte counts, except again for eosinophils. In both sexes, eosinophils decreased during the day, however the drop was larger in females. Eosinophils are associated with clearance of parasitic infections and immune modulation by regulating other immune cells (e.g., T-cells, B-cells, neutrophils and basophils). Sex differences in parasite infection are a relatively common phenomenon in a wide range of avian and mammalian species, with males usually having higher parasite prevalence and abundance compared to females (Christe et al., 2007; Pap et al., 2010). In several bat species, however, higher parasitism was reported for various ectoparasites (mites, ticks, bat flies) in females (e.g., Sharifi et al., 2008; Postawa & Szubert-Kruszyńska, 2014; Patterson, Dick & Dittmar, 2008) due to the sex-specific social and spatial segregation in these species (Christe et al., 2007). Although we have no information on parasite diversity and abundance of *C. plicatus,* female-biased ectoparasitism might explain the higher eosinophil numbers in females. Additionally, it has been shown that ectoparasites survive better on adult females than on adult males (Christe et al., 2007). Although eosinophils may play only a minor role in LPS response compared to, for example, neutrophils, it has been shown that intradermal injection of LPS leads to a marked and dose-dependent accumulation of eosinophils into guinea-pig skin sites (Weg et al., 1995). Additive effects of LPS challenge and the higher parasites in experimental females might explain the lower diurnal decrease in eosinophils compared to the experimental males and control bats.

## Conclusions

In short, our findings suggest that *C. plicatus* exhibited a circadian rhythm in immune cells. Therefore, we conclude that the timing of bleeding is essential when comparing blood cell counts across species. Further, diurnal fluctuations of immune cells might correspond to the diurnal changes in the relative risk of infection. The magnitude of these fluctuations may vary across bats according to specific-specific infection risks associated with colony sizes or specific roosting habits. LPS challenges induced a cellular immune response in *C. plicatus* which either led to an increase in immune cells or to constant levels during the 8 h treatment period, as opposed to the decreasing levels for almost all immune cells observed in control animals. Further studies are needed to provide more fine-scaled information on the temporal dynamics of immune cells and the relative immunological response of bats when responding to bacterial infections at different times of the day.

##  Supplemental Information

10.7717/peerj.3570/supp-1 Data S1 Hematological data Raw hematological data of *Chaerephon plicatus* according to treatment (PBS or control) before (A) and after (B).

## Additional Information and Declarations

### Data Availability

The following information was supplied regarding data availability: The raw data has been supplied as Data S1.
